# Association between physical activity and bone mineral density in postmenopausal women: a cross-sectional study from the NHANES 2007–2018

**DOI:** 10.1186/s13018-023-03976-2

**Published:** 2023-07-15

**Authors:** Jiazhong Ji, Yue Hou, Zhaoyang Li, Ying Zhou, Huaming Xue, Tao Wen, Tao Yang, Long Xue, Yihui Tu, Tong Ma

**Affiliations:** 1 Department of Orthopaedics, Yangpu Hospital, School of Medicine, Tongji University, Shanghai, 200090 China; 2 Department of Obstetrics and Gynecology, Qilu Hospital of Shandong University, Jinan, Shandong China; 3 Shidong Hospital, Yangpu District Shidong Hospital affiliated to University of Shanghai for Science and Technology, 999 Shiguang Road, Shanghai, 200438 China

**Keywords:** Physical activity, Bone mineral density, Postmenopausal women, NHANES

## Abstract

**Background:**

Physical activity (PA) is generally encouraged for the treatment of osteoporosis. However, epidemiological statistics on the level of physical activity required for bone health are scarce. The purpose of this research was to analyze the association between PA and total spine bone mineral density (BMD) in postmenopausal women.

**Methods:**

The research study included postmenopausal women aged ≥ 50 from the National Health and Nutrition Examination Survey. The metabolic equivalent (MET), weekly frequency, and duration of each activity were used to calculate PA. Furthermore, the correlations between BMD and PA were investigated by multivariable weighted logistic regression.

**Results:**

Eventually, 1681 postmenopausal women were included, with a weighted mean age of 62.27 ± 8.18 years. This study found that performing ≥ 38MET-h/wk was linked to a lower risk of osteoporosis after controlling for several covariates. Furthermore, the subgroup analysis revealed that the connection between total spine BMD and moderate-to-vigorous PA was more obvious among postmenopausal women aged < 65 years or individuals with normal BMI (< 25 kg/m^2^).

**Conclusion:**

Physical activity ranging from moderate to vigorous was linked to higher total spine BMD in postmenopausal women.

**Supplementary Information:**

The online version contains supplementary material available at 10.1186/s13018-023-03976-2.

## Introduction

Osteoporosis after menopause and brittle fractures are serious worldwide health concerns [1, 2]. Osteoporosis affects 200 million people worldwide, and 8.9 million fractures happen each year [3]. Hip fracture cases could top 21 million by 2050 [4]. Globally, the prevalence of osteoporosis is 18.3%, with women being more affected than men [5]. The lifetime probability of experiencing an osteoporotic hip, spine, as well as forearm fracture in white women is estimated to be 40–50% [6, 7]. Therefore, avoiding and treating osteoporosis in patients with osteoporosis and osteopenia is crucial. Other aspects of bone metabolism, such as lipid metabolism and exercise, have recently gotten lots of focus in addition to genetics, age, and gender [8–13].

Physical activity (PA) has been demonstrated to perform a significant role in improving quality of life by preventing or treating degenerative diseases associated with the aging process [14–17]. In middle-aged and older individuals, PA can effectively reduce bone loss and boost bone density [18]. According to a meta-analysis, physical activity can successfully slow or even reverse the decrease of BMD in osteoporotic elderly individuals [19]. Furthermore, the most prevalent poor health consequences related to sarcopenia, osteoporosis, and osteoarthritis can be decreased with PA [15, 16, 19, 20]. However, few have investigated the incidence among varied PA levels in postmenopausal females. As a result, it is critical to investigate the connection between PA levels and BMD to observe whether PA levels can be used to prevent osteoporosis or osteopenia. This investigation may also provide a unique theoretical structure for figuring out the cause of the sickness and developing treatments.

Eventually, we investigated the relationship between PA levels and total spine BMD in the present research with a representative cohort of postmenopausal women aged 50 years from the National Health and Nutrition Examination Survey (NHANES).

## Methods

### Data source and study population

The National Health and Nutrition Examination study (NHANES) is a large, continuing cross-sectional study conducted in the USA with the goal of providing objective health statistics and addressing developing public health challenges among people in general. The survey techniques were approved by the National Center for Health Statistics Institutional Review Board, and all NHANES participants allowed for their data to be used in the research. We used the NHANES 2007–2018 data (2007–2008, 2009–2010, 2013–2014, and 2017–2018). The inclusion criteria were as follows: (i) postmenopausal women aged ≥ 50 years and (ii) participants with complete BMD and PA data. The following were the exclusion criteria: (i) participants who were pregnant, (ii) participants with cancer diagnoses, and (iv) participants with a history of using feminine hormones.

### Menopausal status definitions

The state of menopause was determined using a self-reported reproductive health questionnaire. Women were considered to be postmenopausal who responded “no” to the inquiry “Have you had at least one menstrual period in the past 12 months?” and responded “hysterectomy” as well as “menopause/change of life” to the inquiry “What is the reason that you have not had a period in the past 12 months?”. The self-reported reproductive health questionnaire is described in detail on the NHANES website.

### Variables

The Global Physical Activity Questionnaire (GPAQ), which contains questions about daily activities, leisure activities, and sedentary activities, was used to measure individuals’ physical activity (the exposure variable) between 2007 and 2018 [21, 22]. Using the proposed MET score (MET-h/week), PA was then divided into three levels (low, moderate, and high) [23–25]. A Hologic QDR 4500A device and Apex software version 3.2 were used by certified radiology technologists to perform dual-energy X-ray absorptiometry in order to determine total spine BMD. The NHANES database’s covariate selection was based on findings from previous studies [26]. Finally, age, race, body mass index (BMI), a ratio of family income to poverty, education level, diabetes, high blood pressure, smoked at least 100 cigarettes in life, alkaline phosphatase (ALP), aspartate transaminase (AST), alanine transaminase (ALT), blood urea nitrogen, and total calcium were assessed to be potential confounders in this research.

### Statistical analysis

Continuous and categorical variables are presented as mean ± SD and percentage, respectively. In order to examine the connection between PA and total spine BMD, a weighted multivariate logistic regression model was applied. To determine the difference between each group, we utilized the weighted2 test for categorical data and the weighted linear regression model for continuous variables. Subgroup analysis was conducted with stratified factors, including age (< 65; ≥ 65 years), race (non-Hispanic white, non-Hispanic black, Mexican American, and other races), and BMI (normal, overweight, obesity). Both EmpowerStats (version 2.0; http://www.empowerstats.com) and the R program (version 4.0.3; https://www.R-project.org) were used for all analyses. Statistical significance was set at *P* < 0.05.

## Result

### Participant selection and baseline characteristics

**Fig. 1:**
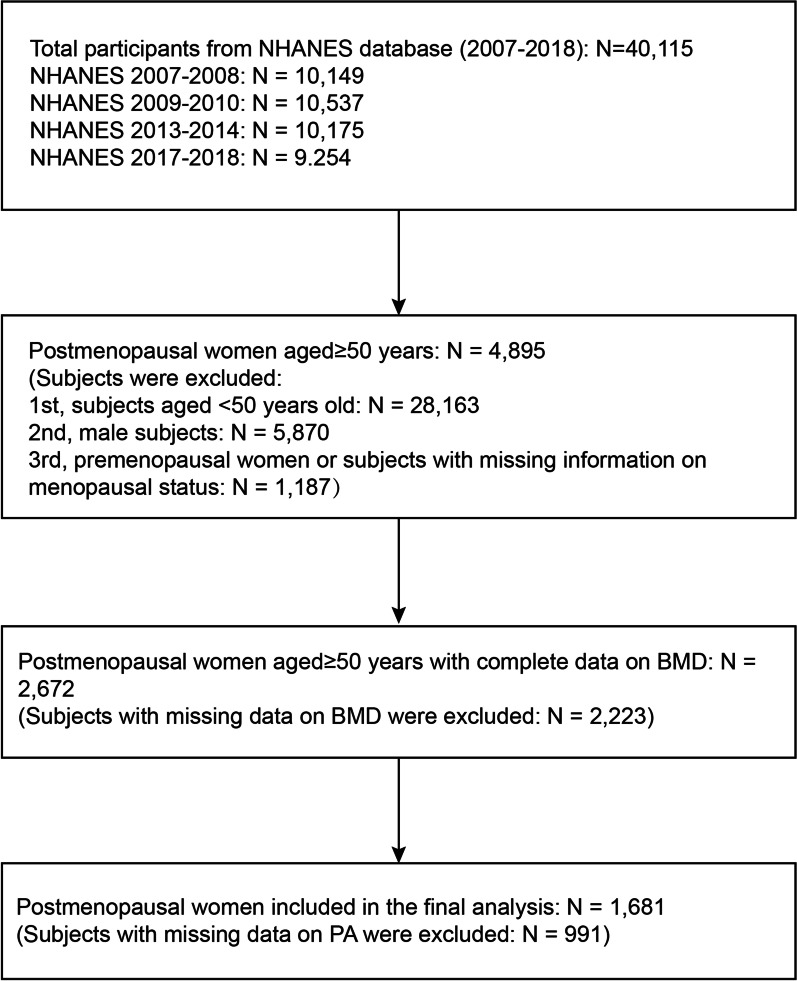
Flowchart of participant selection. BMD, bone mineral density; *NHANES* National Health and Nutrition Examination Survey; and *PA* physical activity

The participant selection flowchart is provided in Fig. 1. Data from 40,115 participants in the NHANES was collected. First, we eliminated people above the age of 50 (N = 28,163) and men (N = 5,870). Premenopausal women and individuals (N = 1,187) who lacked information on their menopausal status were additionally excluded from this study. Furthermore, we eliminated postmenopausal females with incomplete total spine BMD and PA data (N = 2,223 and N = 991, respectively). Eventually, 1681 people were enrolled in the research.

Of the 1682 postmenopausal women enrolled in the research, the mean age at enrollment was 62.27 ± 8.18 years. There were 601 (37.32%) individuals who smoked. In addition, 626 (31.96%) were overweight. More than 281 (11.22%) and 863 (44.64%) had been diagnosed with diabetes and hypertension, respectively. Among all participants, the mean total spine BMD was 0.94 ± 0.15. Based on the level of physical activity among the participants, the weighted features were divided into three tertiles. (Q1: ≤ 11.9MET-h/wk; Q2: 12–37.9MET-h/wk; and Q3: ≥ 38MET-h/wk). The baseline features of the physical activity tertiles differed little. The clinical characteristics of the participants are shown in Additional file 1: Table S1.

Table 1 displays the results of the multivariate regression analysis. When no covariates were adjusted (Model 1), total spine BMD in postmenopausal women did not differ significantly between each group. Postmenopausal women in a higher PA level (Q2 and Q3) had significantly higher total spine BMD than the controls in Model 2, and this difference remained significant in Model 3. Moreover, participants in Q3 had a 0.022 g/cm^2^ higher BMD than those in Q2.

**Table 1:** Association between PA (MET-h/wk) and total spine BMD (g/cm^2^)

	Model 1 β (95%CI) ***P***-value	Model 2 β (95%CI) ***P***-value	Model 3 β (95%CI) ***P***-value
Total spine BMD			
Q1	Reference	Reference	Reference
Q2	0.010 (−0.008,0.028) 0.283	0.017 (0.000,0.033) 0.046	0.017 (0.000,0.034) 0.040
Q3	0.010 (−0.008,0.029) 0.264	0.017 (0.000,0.034) 0.049	0.022 (0.005,0.038) 0.011
p for trend	0.432	0.182	0.048

PA:Q1 (≤ 11.9MET-h/wk), Q2 (12–37.9MET-h/wk), Q3 (≥ 38MET-h/wk)

Model 1: No covariates were adjusted

Model 2: Age, race, and BMI were adjusted

Model 3: Age, race, BMI, ratio of family income to poverty, education level, diabetes, high blood pressure, smoked at least 100 cigarettes in life, ALP, AST, ALT, blood urea nitrogen, and total calcium were adjusted

After adjusting the covariates, the results of subgroup analysis revealed that participants in a higher PA level (Q2 and Q3) had significantly higher BMD than the controls in women aged < 65 years, women with normal BMI (BMI < 25 kg/m^2^), non-Hispanic white women, or women of other ethnicities (race/ethnicity other than non-Hispanic white, non-Hispanic black, or Mexican American). Tables 2, 3, and 4 display the findings of the subgroup analysis broken down by age, race, and BMI.

**Table 2:** Association between PA (MET-h/wk) and total spine BMD (g/cm^2^) stratified by age

	Model 1 β (95%CI) *P*-value	Model 2 β (95%CI) *P*-value	Model 3 β (95%CI) *P*-value
Total spine BMD			
Aged < 65			
Q1	Reference	Reference	Reference
Q2	0.022 (−0.002, 0.045) 0.072	0.034 (0.013, 0.055) 0.002	0.033 (0.012, 0.054) 0.002
Q3	0.009 (−0.014, 0.032) 0.423	0.028 (0.008, 0.049) 0.008	0.032 (0.012, 0.052) 0.002
Aged ≥ 65			
Q1	Reference	Reference	Reference
Q2	−0.008 (−0.037, 0.021) 0.603	−0.005 (−0.032, 0.022) 0.697	−0.008 (−0.035, 0.019) 0.557
Q3	0.009 (−0.022, 0.041) 0.564	0.009 (−0.020, 0.038) 0.544	0.013 (−0.016, 0.042) 0.387

Model 1: No covariates were adjusted

Model 2: Race, and BMI were adjusted

Model 3: Race, BMI, ratio of family income to poverty, education level, diabetes, high blood pressure, smoked at least 100 cigarettes in life, ALP, AST, ALT, blood urea nitrogen, and total calcium were adjusted

**Table 3:** Association between PA (MET-h/wk) and total spine BMD (g/cm^2^) stratified by race

	Model 1 β (95%CI) *P*-value	Model 2 β (95%CI) *P*-value	Model 3 β (95%CI) *P*-value
Total spine BMD			
Non-Hispanic White			
Q1	Reference	Reference	Reference
Q2	0.024 (−0.003, 0.052) 0.077	0.028 (0.003, 0.053) 0.030	0.025 (0.000, 0.049) 0.053
Q3	0.021 (−0.006, 0.048) 0.120	0.025 (0.000, 0.050) 0.048	0.029 (0.004, 0.054) 0.022
Non-Hispanic Black			
Q1	Reference	Reference	Reference
Q2	−0.035 (−0.069, 0.000) 0.052	−0.026 (−0.056, 0.005) 0.096	−0.024 (−0.054, 0.007) 0.127
Q3	−0.042 (−0.077,−0.007) 0.019	−0.034 (−0.065, −0.003) 0.032	−0.028 (−0.059, 0.004) 0.083
Mexican American			
Q1	Reference	Reference	Reference
Q2	−0.009 (−0.058, 0.041) 0.729	−0.003 (−0.048, 0.042) 0.890	−0.005 (−0.049, 0.038) 0.809
Q3	0.002 (−0.045, 0.049) 0.94634	0.009 (−0.034, 0.052) 0.682	0.004 (−0.039, 0.047) 0.855
Other race/ethnicity			
Q1	Reference	Reference	Reference
Q2	−0.030 (−0.078, 0.018) 0.223	−0.007 (−0.053, 0.039) 0.754	0.006 (−0.038, 0.049) 0.802
Q3	0.010 (−0.042, 0.062) 0.71	0.016 (−0.033, 0.066) 0.517	0.016 (−0.030, 0.062) 0.506

Model 1: No covariates were adjusted

Model 2: Age and BMI were adjusted

Model 3: Age, BMI, ratio of family income to poverty, education level, diabetes, high blood pressure, smoked at least 100 cigarettes in life, ALP, AST, ALT, blood urea nitrogen, and total calcium were adjusted

**Table 4:** Association between PA (MET-h/wk) and total spine BMD (g/cm^2^) stratified by BMI

	Model 1 β (95%CI) *P*-value	Model 2 β (95%CI) *P*-value	Model 3 β (95%CI) *P*-value
Total spine BMD			
Normal			
Q1	Reference	Reference	Reference
Q2	0.028 (−0.001, 0.057) 0.060	0.025 (−0.005, 0.054) 0.098	0.025 (−0.004, 0.054) 0.097
Q3	0.051 (0.022, 0.081) 0.001	0.043 (0.013, 0.073) 0.005	0.050 (0.020, 0.079) 0.001
Overweight			
Q1	Reference	Reference	Reference
Q2	−0.001 (−0.029, 0.028) 0.962	−0.001 (−0.030, 0.027) 0.925	0.000 (−0.028, 0.029) 0.984
Q3	0.009 (−0.019, 0.037) 0.523	0.004 (−0.023, 0.032) 0.753	0.006 (−0.022, 0.034) 0.669
Obesity			
Q1	Reference	Reference	Reference
Q2	0.023 (−0.007, 0.052) 0.133	0.019 (−0.010, 0.049) 0.196	0.022 (−0.006, 0.051) 0.128
Q3	−0.012 (−0.042, 0.019) 0.454	−0.017 (−0.047, 0.013) 0.270	−0.001 (−0.031, 0.028) 0.923

Model 1: No covariates were adjusted

Model 2: Age and race were adjusted

Model 3: Age, race, ratio of family income to poverty, education level, diabetes, high blood pressure, smoked at least 100 cigarettes in life, ALP, AST, ALT, blood urea nitrogen, and total calcium were adjusted

## Discussion

In the current study, our multivariate logistic regression research revealed that a higher level of PA was related to a better total spine BMD. Moreover, the subgroup analysis showed that the associations between BMD and the higher PA level were apparent in postmenopausal women aged < 65 years or those with normal BMI (BMI < 25 kg/m^2^).

Our findings on the relationship between BMD and PA levels are consistent with the significant findings in this field compiled in a recent meta-analysis, which shows that proper PA can effectively inhibit or even reverse the decline of BMD in older people with osteoporosis [19]. Although there is heterogeneity among research, the majority of them indicate that weight-bearing and resistance physical activities have a beneficial effect on the prevention and treatment of osteoporosis in elderly people [27–29]. In a randomized controlled research, Watson et al. discovered that high-intensity progressive resistance and impact weight PA was superior than low-intensity home training in terms of improving BMD in the lumbar spine of postmenopausal women [30]. Similar results from a recent review by Mohebbi (2023) demonstrated that exercise has a positive effect on BMD in postmenopausal women [31]. Kim et al. found that moderate-to-vigorous physical activity was positively associated with hip BMD in Korean males, and there was no correlation between PA and BMD at any site in females [32]. This research has some benefits over the preceding investigation [33]. First, previous research focused on the relationship between BMD and PA in Asian ethnicities. Our research participants, however, contrasted from these analyses, and our findings offered additional data on the relationship of total spine BMD with PA among postmenopausal women in the US population. Second, the current study demonstrates a potential dose–response relationship, demonstrating that high levels of PA (38MET-h/wk) are superior to low levels of PA (11.9MET-h/wk) in improving total spine BMD in postmenopausal women. Finally, unlike prior research, this study conducted a subgroup analysis to assess how additional factors might affect the connection between PA and total spine BMD.

The current research not only demonstrates a connection between PA and total spine BMD in postmenopausal women but also significant ramifications for doctors. A higher PA level may likewise be associated with a higher BMD due to the correlation’s positive nature. In our research, we also used subgroup analysis to get a more accurate picture of the data set. We discovered that the connection between total spine BMD and PA was stronger in postmenopausal women over the age of 65. Furthermore, divided based on ethnicity and BMI, our subgroup analyses discovered a more significant association between PA and BMD among postmenopausal women with normal BMI (BMI 25 kg/m^2^), non-Hispanic white women, or women of other ethnicities for the first time. Differences in levels of estrogen, genetic susceptibility, environmental risk factors, and other factors may explain BMI and race disparities. More additional research with large cohort populations is needed to better understand the relationship between PA and BMD by BMI and race.

The mechanisms underlying the link between PA and total spine BMD are unknown. There is no compelling evidence to endorse this negative connection, particularly in basic research. Osteoblasts are well-known mechanical receptors that can convert mechanical stimuli into biochemical signals that promote bone matrix formation and mineralization [34–37]. Moreira et al. conducted a high-intensity physical activity program and discovered that it was effective in enhancing the formation marker P1NP while simultaneously decreasing the growth of bone resorption marker [38]. Mechanical strain promotes osteoblast matrix mineralization [34, 39] and increases the expression of osteoblast ECM-related proteins such as osteonectin, osteopontin (OPN), osteocalcin (OCN), bone morphogenetic protein 2 (BMP-2), and type I collagen [39]. Furthermore, mechanical strain promotes the synthesis of matrix-bound vascular endothelial growth factor (mVEGF), which has angiogenic properties in vivo [40, 41]. The biomechanics of bones can be regulated by PA, which can also stress bones mechanically and promote osteoblast differentiation and bone cell formation. Moreover, local blood circulation in the bones boosts metabolism, enhances bone cell activity, lowers bone turnover, and restores bone structure and bone mass as a result of PA [42–44]. Since basic research on how PA affects BMD is sparse, future studies should focus on the particular processes by which elevated PA levels alter bone metabolism, which is required to expand theoretical knowledge of the effects of PA on bone homeostasis.

## Strength and study limitation

Many randomized controlled and cohort studies to date have concentrated on postmenopausal women [45–48]. Since we chose a nationally representative sample, the results of our research are quite applicable to the overall population. While finding a sizable modification of PA on total spine BMD, numerous limitations were unavoidable. First of all, because this analysis is cross-sectional, it is challenging to draw conclusions about causality, which is a natural limitation of ecological research. Second, there were too many covariates to control for them all. For instance, hyperthyroidism is a significant factor affecting osteoporosis, but it was not possible to include it in the analysis since the database did not contain the relevant diagnosis. Although we discussed biological justifications in the discussion section, future cohort studies, and animal tests need to be improved to validate our findings. A plausible conclusion from our studies is that PA should be regarded conventional treatment for osteoporosis, even though there is inadequate data to establish the impact of PA intensity on total spine BMD when combined with antiresorptive bone medication.

The impact of high-intensity PA on bone strength indicators, including bone structural metrics, should be further investigated using additional substantial RCTs with excellent methodology.

## Conclusions

This study shows that PA levels are positively associated with total spine BMD in postmenopausal women, and that high levels of PA significantly increase total spine BMD. We provide further evidence that PA has a positive effect on total spine BMD in postmenopausal women, and the findings of this study offer some crucial references for clinicians and researchers on therapeutic exercise treatment approaches. To further understand the role of PA in osteoporosis, however, due to the inherent limitations of the current research, further large-scale investigations will be necessary.

## Supplementary Information

**Additional file 1**: **Table S1** Characteristics of participants. %, weighted proportion. *ALP* alkaline phosphatase; *AST* aspartate aminotransferase; *ALT* alanine aminotransferase; BMI, body mass index; normal, BMI < 25 kg/m^2^; overweight, 25 ≤ BMI < 30 kg/m^2^; obesity, BMI ≥ 30 kg/m^2^.

## Data Availability

The survey data are publicly available on the Internet for data users and researchers throughout the world (www.cdc.gov/nchs/nhanes/).
