# Supplementary material for: Association between physical activity and bone mineral density in postmenopausal women: a cross-sectional study from the NHANES 2007–2018
Source: J Orthop Surg Res. 2023 Jul 15;18:501. doi: 10.1186/s13018-023-03976-2 (PMC10349409; doi:10.1186/s13018-023-03976-2)
Supplement: Supplementary file 1 — Additional file 1: Table S1 Characteristics of participants. %, weighted proportion. ALP alkaline phosphatase; AST aspartate aminotransferase; ALT alanine aminotransferase; BMI, body mass index; normal, BMI < 25 kg/m2; overweight, 25 ≤ BMI < 30 kg/m2; obesity, BMI ≥ 30 kg/m2. [file 13018_2023_3976_MOESM1_ESM.docx]

**Table S1 Characteristics of participants**

| **Characteristics** | **Total (N=1682)** | **Q1，≤11.9MET-h/wk**  **(N=485)** | **Q2，12-37.9MET-h/wk**  **(N=632)** | **Q3，≥38MET-h/wk**  **(N=565)** | ***P*-value** |
| --- | --- | --- | --- | --- | --- |
| Age (years, mean ± SD) | 62.27 ± 8.18 | 64.49 ± 8.69 | 63.84 ± 8.04 | 61.74 ± 7.80 | <0.001 |
| Race/ethnicity (n, %) |  |  |  |  | 0.418 |
| Non-Hispanic White | 762(73.97%) | 212 (43.71%) | 298 (47.15%) | 252 (44.60%) |  |
| Non-Hispanic Black | 514(14.64%) | 151 (31.13%) | 184 (29.11%) | 179 (31.68%) |  |
| Mexican American | 234(4.59%) | 70 (14.43%) | 78 (12.34%) | 86 (15.22%) |  |
| Other race/ethnicity | 172(6.80%) | 52 (10.72%) | 72 (11.39%) | 48 (8.50%) |  |
| BMI status,n (%) |  |  |  |  | 0.148 |
| normal | 495(35.75%) | 139 (28.66%) | 201 (31.80%) | 155 (27.43%) |  |
| overweight | 561(32.29%) | 149 (30.72%) | 207 (32.75%) | 205 (36.28%) |  |
| obesity | 626(31.96%) | 197 (40.62%) | 224 (35.44%) | 205 (36.28%) |  |
| Ratio of family income-to-poverty | 3.25 ± 1.56 | 2.64 ± 1.52 | 2.86 ± 1.61 | 2.73 ± 1.51 | 0.071 |
| Education Level (n, %) |  |  |  |  | 0.010 |
| Less than high | 372(13.15%) | 127 (26.19%) | 125 (19.78%) | 120 (21.24%) |  |
| school High school | 413(26.38%) | 118 (24.33%) | 139 (21.99%) | 156 (27.61%) |  |
| More than high school | 897(60.47%) | 240 (49.48%) | 368 (58.23%) | 289 (51.15%) |  |
| Diabetes (n, %) |  |  |  |  | 0.025 |
| Yes | 281(11.22%) | 102 (21.03%) | 100 (15.82%) | 79 (13.98%) |  |
| No | 1344(85.42%) | 362 (74.64%) | 511 (80.85%) | 471 (83.36%) |  |
| Borderline | 58(3.36%) | 21 (4.33%) | 21 (3.32%) | 16 (2.66%) |  |
| High blood pressure (n, %) |  |  |  |  | 0.083 |
| Yes | 863(44.64%) | 264 (54.43%) | 329 (52.06%) | 270 (47.79%) |  |
| No | 819(55.36%) | 221 (45.57%) | 303 (47.94%) | 295 (52.21%) |  |
| Smoked at least 100 cigarettes in life,n (%) |  |  |  |  | 0.428 |
| Yes | 601(37.32%) | 184 (37.94%) | 216 (34.18%) | 201 (35.58%) |  |
| No | 1081(62.68%) | 301 (62.06%) | 416 (65.82%) | 364 (64.42%) |  |
| ALP(IU/L, mean ± SD) | 74.40 ± 23.42 | 76.90 ± 26.50 | 73.88 ± 22.43 | 78.17 ± 25.11 | 0.008 |
| AST(U/L,mean ±SD) | 23.99 ± 8.43 | 24.49 ± 11.04 | 24.85 ± 9.64 | 24.35 ± 9.28 | 0.672 |
| ALT(U/L,mean ±SD) | 21.83 ± 10.97 | 21.98 ± 11.61 | 22.64 ± 12.69 | 22.34 ± 13.71 | 0.693 |
| Blood urea nitrogen(mg/dL, mean±SD) | 14.52 ± 5.04 | 14.45 ± 5.13 | 14.82 ± 5.90 | 14.44 ± 5.08 | 0.382 |
| Total calcium (mg/dL, mean±SD) | 9.47 ± 0.37 | 9.47 ± 0.38 | 9.47 ± 0.37 | 9.49 ± 0.36 | 0.822 |
| Total spine BMD (g/cm2, mean ± SD) | 0.94 ± 0.15 | 0.93 ± 0.16 | 0.94 ± 0.16 | 0.94 ± 0.15 | 0.500 |

%, weighted proportion.

ALP, alkaline phosphatase; AST, aspartate aminotransferase; ALT, alanine aminotransferase; BMI, body mass index; normal, BMI<25 kg/m2; overweight, 25≤BMI<30 kg/m2; obesity, BMI≥30 kg/m2;
